# Come in from the Cold: Are Older Adults Who Live in Colder Climates at Greater Risk for Sarcopenia?

**DOI:** 10.3390/jcm9061859

**Published:** 2020-06-15

**Authors:** David Scott

**Affiliations:** 1 Department of Medicine, School of Clinical Sciences at Monash Health, Monash University, Clayton, VIC 3168, Australia; david.scott@monash.edu; Tel.: +61-3-8572-2397; 2 Institute for Physical Activity and Nutrition, School of Exercise and Nutrition Sciences, Deakin University, Burwood, VIC 3125, Australia; 3 Department of Medicine and Australian Institute of Musculoskeletal Science, Melbourne Medical School–Western Campus, The University of Melbourne, St Albans, VIC 3021, Australia

The operational definition of “sarcopenia”, an age-related skeletal muscle disease resulting from adverse changes that accrue across the lifetime, was recently updated by the European Working Group on Sarcopenia in Older People (EWGSOP) [1]. The revised definition differs from the original in terms of suggested measurements, cut-off points and case-finding algorithms. Consequently, reported sarcopenia prevalence estimates have drastically changed since the publication of this definition [2] and further investigation of the impacts of the revision are warranted in diverse populations.

A recent article by Su et al., published in the Journal of Clinical Medicine, explores the prevalence of sarcopenia in older adults residing on the northern island of Hokkaido, Japan, which is described by the authors as “cold and snowy in the winter, with the roads covered with snow from November to April” [3]. This in an interesting population in which to investigate sarcopenia prevalence given that older adults living in colder climates may have increased likelihood for sarcopenia.

There are several mechanisms by which residing in colder climates may exacerbate age-related muscle declines. In particular, it has been hypothesised that the presence of snow and icy conditions may increase older adults’ fear of falling and subsequently restrict physical activity [4]. A study of community-dwelling Scottish older adults, however, reported that presence of snow did not appear to influence physical activity, despite the fact that colder temperatures and shorter days are associated with reduced levels of activity [5]. On the other hand, presence of ice affects the way almost half of US adults go about their daily activities, and compared with those under the age of 65 years, older adults are twice as likely to report great difficulty in leaving the home in these conditions [6]. Living at higher latitudes is also associated with having lower vitamin D levels, although colder seasons may have a stronger negative influence than latitude [7]. Higher vitamin D levels are consistently associated with better muscle function [8,9] in older adults and so those living in colder climates may have greater risk for hypovitaminosis D-related functional declines.

A study of community-dwelling older adults at increased risk of falls has reported that sarcopenia prevalence estimates may range from 3% to 26%, depending on which of the revised EWGSOP definition’s recommended measurements are used [10]. Thus, it is difficult to compare the results of studies that have utilised different measurements to assess muscle mass and function even when the same operational definition is applied. These challenges are compounded by the fact that age-related declines in muscle mass and function may vary by ethnicity within and between countries [11,12], and that recommended cut-points for sarcopenia, which have generally been derived from Caucasian populations, are generally not appropriate for other ethnicities [1]. These ethnic differences in muscle mass and function need to be taken into account when exploring whether there is indeed an effect of climate on sarcopenia prevalence in older adults. 

Using the EWGSOP’s revised algorithm, and measurements including hand grip strength and muscle quantity assessed by bioelectrical impedance analysis (BIA), Su et al. report the prevalence of sarcopenia in 310 adults aged 65 years and older residing in Hokkaido to be 8.1% [3]. Interestingly, a recent study of older adults residing on the southern Japanese island of Kyushu [13] reported that sarcopenia prevalence, determined using similar methods to identify sarcopenia cases, was between 6.6% and 7.4%, which is similar to that observed by Su et al., for the northern island of Hokkaido. The prevalence reported in these Japanese populations is much higher than we previously observed (1.9%) using the revised EWGSOP definition in a large population of 70-year-olds residing at a much higher latitude (63°), in Umea, Sweden, compared with Hokkaido (43°) [14], but similar to that reported in a study of older adults in Liège, Belgium (7.4%) which is also at a higher latitude (50°) than Hokkaido [2]. Taken together, these studies of sarcopenia, utilising current case-finding guidelines, do not appear to indicate a latitudinal difference in prevalence amongst community-dwelling older adults.

While it does not appear that sarcopenia prevalence is greater for older adults in colder climates, clinicians should recognise that individuals living in these regions may face greater barriers to adopting and maintaining lifestyle behaviours that promote healthy ageing. Older adults should be reminded that the approach with the strongest evidence for improving muscle mass, strength and function is accessible in any climate; progressive resistance training, such as weight-lifting which can be performed in indoor gymnasiums and clinics, is recommended as the first line therapy to manage sarcopenia [15]. Clinicians can support patients with sarcopenia to engage in individually-tailored resistance training programs by referral to qualified professionals such as exercise physiologists.
